# A Facile Synthesis of α-*N*-Ribosyl-Asparagine and α-*N*-Ribosyl-Glutamine Building Blocks

**DOI:** 10.3390/molecules18088779

**Published:** 2013-07-24

**Authors:** Gaetano Speciale, Anna Bernardi, Filippo Nisic

**Affiliations:** Universita’ degli Studi di Milano, Dipartimento di Chimica, via Golgi 19, 20133 Milano, Italy; E-Mails: g.speciale@student.unimelb.edu.au (G.S.); anna.bernardi@unimi.it (A.B.)

**Keywords:** ADP-ribosylation, glycoconjugates, ribofuranosyl aminoacids, Staudinger ligation, stereoselective synthesis

## Abstract

Adenosine diphosphate ribosylation (ADP-ribosylation) is a widely occurring post-translational modification of proteins at nucleophilic side chain of amino acid residues. Elucidation of ADP-ribosylation events would benefit greatly from the availability of well-defined ADP-ribosylated peptides and analogues thereof. In this paper we present a novel approach to the chemical synthesis of ribosylated amino acid building blocks using traceless Staudinger ligation. We describe an efficient and stereoselective synthesis of α-*N*-ribosyl-asparagine (α-*N*-ribosyl-Asn) and α-*N*-ribosyl-glutamine (α-*N*-ribosyl-Gln) building blocks starting from 5-*tert*-butyldiphenylsilyl-β-d-ribofuranosyl azide. The *N*-glycosyl aminoacids are produced in good yields as pure α-anomers, suitably protected for peptide synthesis.

## 1. Introduction

**Scheme 1:**
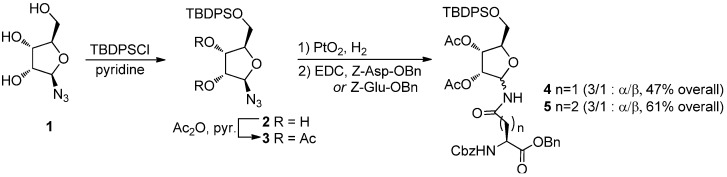
Filippov’s synthesis of ribosylated amino acids (from ref. [8]).

Glycosylated peptides play a fundamental role in biological systems: in fact, more than half of all proteins carry carbohydrate moieties, generating different glycoforms whose exact composition often controls protein function and distribution in biological systems [1,2,3,4]. The details of glycan regulation of protein activity and stability are still under intense scrutiny and the synthesis of well-defined glycopeptides is therefore an important target [5,6], which still presents many challenges to organic chemistry. One of the processes that is currently being elucidated is adenosine diphosphate ribosylation (ADP-ribosylation), a wide-occurring post-translational modification effected by enzymes that transfer ADP-ribose from NAD^+^ to Asn, Glu, Asp, Arg or Cys residues of proteins, so altering their function [7]. An important contribution for the clarification of the role of ADP-ribosylation events came in 2010, when Filippov and coworkers [8] reported the synthesis of ribosylated oligopeptides using α-*N*-ribosyl-asparagine (α-*N*-ribosyl-Asn) and α-*N*-ribosyl-glutamine (α-*N*-ribosyl-Gln) building blocks. Despite the relevance of this work, the approach suffers from a poorly selective synthesis of the required ribosylated building blocks **4**α and **5**α (Scheme 1), which were prepared by PtO_2_ reduction of azide **3**, followed by EDC-mediated coupling of the resulting mixture of epimeric amines with Z-Asp-OBn or Z-Glu-OBn, respectively. The *N*-ribosyl-aminoacids were obtained as a 3:1 α: β anomeric mixtures, from which the desired α-anomers were to be chromatographically isolated. Acetyl transfer to the anomeric nitrogen from the 2-*O*-acetate also occurs during the reaction, further reducing the process yields, particularly for the asparagine derivative **4**α. 

**Scheme 2:**
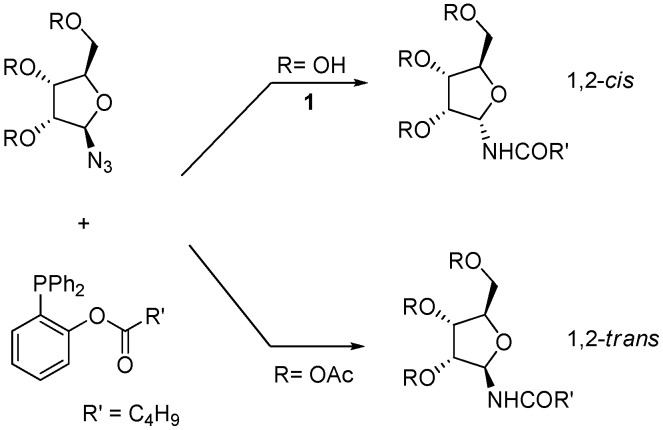
Traceless Staudinger ligation of furanosyl azides with functionalized phosphines (from ref [9]).

We have recently developed a protocol for the stereoselective synthesis of either α- or β-*N*-glycofuranosyl amides using the Staudinger traceless ligation [9,10]. In this reaction, a starting anomeric glycosyl azide is partially reduced by an appropriate phosphine and then intramolecularly acylated in order to afford the amide (Scheme 2) [11]. We have shown that for furanosyl azides the outcome of the process is controlled by the configuration and the protection state of the contiguous hydroxyl group, so that 1,2-*cis* amides (α-, in the *ribo* series) are obtained when this group is unprotected and 1,2-*trans* amides (β-, in the *ribo* series) when it is acetylated. For unprotected furanoses, the anomeric configuration of the product amides appears to be dictated by O-P coordination, as supported by ^31^P-NMR studies [10].

As an application of this methodology, we here report the facile stereoselective synthesis of **4α **and **5α** that could be obtained in good yields and with full stereoselectivity with a single synthetic operation starting from β-5-*O*-*tert-*butyldiphenylsilyl-ribosylazide **2**.

## 2. Results and Discussion

**Scheme 3:**
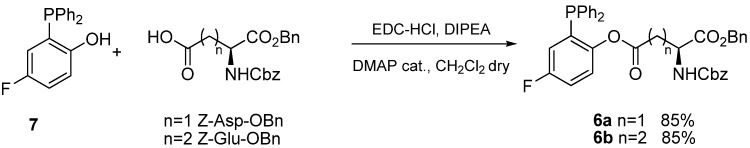
Synthesis of functionalized phosphines **6a** and **6b**.

Previous research in our laboratories had shown that transfer of aminoacid chains in Staudinger ligations of glycosyl azides was particularly effective using fluorinated phosphines **6** [12]. Thus we envisaged that phosphines **6a** and **6b** (Scheme 3), functionalized with Z-Asp-OBn and Z-Glu-OBn acyl chains, respectively, would be the reagent of choice to prepare the target α-*N*-ribosyl-Asn and α-*N*-ribosyl-Gln building blocks. Phosphines **6a** and **6b** were synthesized in excellent yields by EDC-mediated acylation of 2-diphenylphosphanyl-5-fluorophenol (**7**) [13] and purified by filtration on short pads of silica. 

**Scheme 4:**
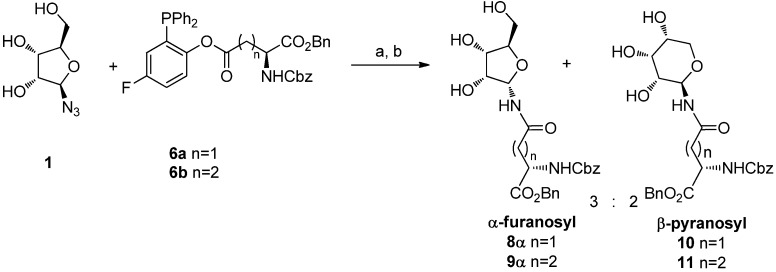
Ligation of **1** with phosphines **6a** and **6b**.

An early trial of ligation was conducted using the unprotected β-d-ribofuranosyl azide **1** [14] with phosphines **6a** and **6b** for 20 h at 70 °C in a 98:2 DMA:DMPU [15] solution, followed by water quenching (Scheme 4). In both ligation reactions 45% of a 3:2 mixture of α-ribofuranosyl amide (**8**α or **9**α) and β-ribopyranosyl amide (**10** or **11**) was obtained. The isomer distribution was determined by ^1^H-NMR analysis of the crude reaction mixtures: the α-ribofuranosyl amide (**8**α or **9**α) was characterized by the presence of the H1 proton signal at 5.65 ppm and by the high chemical shift of C4 (84.3 ppm), which is diagnostic for the furanose form. The structures of **10** and **11** were assigned on the basis of the vicinal coupling constant of the anomeric protons (*J_1,2_* = 8 Hz, H1 = 5.12 ppm) and of the low chemical shift value of C4 (68.8 ppm), that confirms the pyranose structure. The β-glycopyranosyl isomers **10** and **11** obtained in these reactions must derive from ring-expansion occurring after a ring-opening process.

*Reagents and Conditions*: DMA/DMPU 98:2, 70 °C, 20 h; (b) H_2_O, 2 h, 70 °C.

**Scheme 5:**
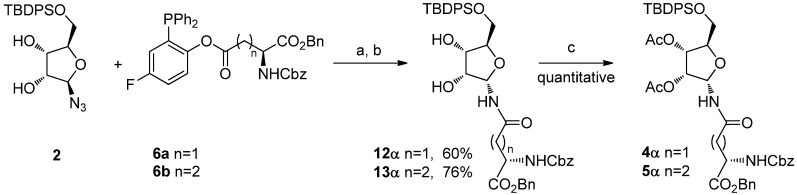
Synthesis of ribofuranosyl amides **4**α and **5**α.

To avoid ring expansion, the 5-hydroxy group of **1** was protected as a *tert-*butyldiphenylsilyl ether and the 5-*tert*-butyldiphenylsilyl-β-D-ribofuranosyl azide** 2** was synthesized in good yields as described by Filippov [8]. Ligation of **2 **with **6a **or** 6b **was carried out as above (20 h at 70 °C in a 98:2 DMA: DMPU mixture) (Scheme 5). In both cases, 400 MHz ^1^H-NMR analysis of the crude showed only one signal in the furanose anomeric region, implying that no pyranose was formed, as expected, and that the α/β ratio of the resulting ribofuranosyl amides **12**α and **13**α was ≥ 99:1. The two compounds were isolated by flash chromatography, which afforded the desired building blocks in 60% and 76% yields, respectively, as single α isomers. After acetylation (Ac_2_O, cat. DMAP, CH_2_Cl_2_, quant.), **4**α and **5**α were obtained. Their spectroscopic data were fully consistent with the already published characterization [8]. 

*Reagents and Conditions*: DMA/DMPU 98:2, 70 °C, 20 h; (b) H_2_O, 2 h, 70 °C; (c) Ac_2_O, cat. DMAP, CH_2_Cl_2_.

## 3. Experimental

Solvents were dried by standard procedures: dichloromethane and *N*,*N*-diisopropylethylamine were dried over calcium hydride; *N,N*-dimethylacetamide (DMA), 1,3-dimethyltetrahydro-2(1*H*)-pyrimidinone (DMPU), chloroform and pyridine were dried over activated molecular sieves. Reactions requiring anhydrous conditions were performed under nitrogen. ^1^H-, ^13^C- and ^31^P-NMR spectra were recorded at 400 MHz on a Bruker AVANCE-400 instrument. Chemical shifts (*δ)* for ^1^H and ^13^C spectra are expressed in ppm relative to internal Me_4_Si as standard. Chemical shifts (*δ)* for ^31^P are expressed in ppm relative to internal H_3_PO_4_ as standard. Signals were abbreviated as s, singlet; bs, broad singlet; d, doublet; t, triplet; q, quartet; m, multiplet. Mass spectra were obtained with a Bruker ion-trap Esquire 3000 apparatus (ESI ionization) or FT-ICR Mass Spectrometer APEX II & Xmass software (Bruker Daltonics)—4.7 Magnet and Autospec Fission Spectrometer (FAB ionization). Thin layer chromatography (TLC) was carried out with pre-coated Merck F_254_ silica gel plates. Flash chromatography (FC) was carried out with Macherey-Nagel silica gel 60 (230–400 mesh). The typical scale used for the ligation reactions in this paper was 20 mg (0.1 mmol) of unprotected glycosyl azide.

### General Procedure for the Synthesis of 2-(diphenylphosphanyl)-4-fluorophenyl Esters **6**

A solution of the *o-*diphenylphosphinophenol (**7**, 1 equiv.), the commercially available *N*-carbobenzyloxy-L-aminoacid-1-benzyl ester (Z-Asp-OBn or Z-Glu-OBn, 1.2 equiv.) and *N,N*-dimethylaminopyridine (0.1 equiv.) in dry CH_2_Cl_2_(0.1 M) were added, at room temperature and under nitrogen, to a suspension of *N*-(3-dimethylaminopropyl)-*N’*-ethylcarbodiimide hydrochloride (EDC, 1.4 equiv.) and dry *N,N*-diisopropylethylamine (1.4 equiv.) in dry CH_2_Cl_2_. The mixture was stirred at RT for 2 h, monitoring by TLC (60:40 hexane/AcOEt). The reaction mixture was diluted with CH_2_Cl_2_ and washed with 5% aqueous HCl and water: the organic layer was dried over Na_2_SO_4_ and concentrated. The crude product obtained was purified by flash chromatography, as indicated in each case.

*1-Benzyl 5-[2-(Diphenylphosphanyl)-4-fluorophenyl] N-(Benzyloxycarbonyl)-l-aspartate* (**6a**). The crude product was purified by flash chromatography (hexane/AcOEt 60:40). yield = 85%. ^1^H-NMR (CDCl_3_, 25 °C): *δ* = 7.37–7.23 (m, 20H, Ph), 7.02–6.84 (m, 2H, H-2, H-3), 6.49 (m, 1H, H-1), 5.69 (d, *J*_NH-CH_ = 8.6 Hz, 1H, NH), 5.17 (s, 2H, CH_2_O), 5.13 (s, 2H, CH_2_O), 4.62 (m, *J*_CH-CH2_ = 4.4 Hz, 1H, CH), 3.00 (dt, *J* = 4.9 Hz, *J* = 17.4 Hz, 1H, H_a_, CH_2_), 2.67 (dd,* J* = 4.3 Hz, *J* = 17.4 Hz, 1H, H_b_, CH_2_). ^13^C-NMR (CDCl_3_, 25 °C): *δ* = 170.4, 169.2, 161.9, 159.4 (CO_(Cbz)_), 134.6, 134.0, 132.0, 131.7, 129.0, 128.9, 128.7, 128.6, 128.4, 128.3, 128.2 (C_Ar_), 124.0, 116.9, 116.6, 67.8 (CH_2_O), 67.3 (CH_2_O), 50.4 (CH), 36.5 (CH_2_). ^31^P-NMR (161 MHz, CDCl_3_^,^ 25 °C):* δ* = −14.3 ppm. FAB-MS: *m/z* 636 (M+1).

*1-Benzyl 5-[2-(Diphenylphosphanyl)-4-fluorophenyl] N-(Benzyloxycarbonyl)-l-glutamate* (**6b**). The crude product was purified by flash chromatography (hexane/AcOEt 70:30). yield = 85%. ^1^H-NMR (CDCl_3_, 25 °C): *δ* = 7.37–7.24 (m, 20 H, Ph), 7.05 (m, 1H, H-2), 6.99 (m, 1H, H-3), 6.45 (m, 1H, H-1), 5.37 (d, *J*_NH-CH_ = 8.1 Hz, 1H, NH), 5.16 (s, 2H, CH_2_-O), 5.07 (s, 2H, CH_2_-O), 4.38 (m, 1H, CH), 2.26 (m, 2H, CH_2_-CO), 2.05 (m, 1H, H_a_, CH_2_-CH), 1.80 (m, 1H, H_b_, CH_2_-CH). ^13^C-NMR (CDCl_3_, 25 °C): *δ* = 170.7, 161.7, 159.3, 156.1 (CO_(Cbz)_), 134.4, 134.3, 134.2, 134.0, 129.1, 129.0, 128.8, 128.5, 128.4 (C_Ar_), 124.2, 124.1, 120.0, 119.8, 116.8, 116.5, 67.6 (CH_2_-O), 67.4 (CH_2_-O), 53.5 (CH), 30.1 (CH_2_-CO), 27.4 (CH_2_). ^31^P-NMR (161 MHz, CDCl_3_, 25 °C):* δ* = −14.3 ppm. FAB-MS: *m/z* 650 (M+1).

### General Procedure for the Staudinger Traceless Ligation of Ribofuranosyl Azides

Phosphine **6a** or **6b** (2 equiv.) was added, at room temperature, to a 0.1 M solution of ribofuranosyl azide **2 **(1 equiv.) in 98: 2 *N,N*-dimethylacetamide and DMPU. The solution was stirred for 20 h at 70 °C, then 10% water was added and the mixture was stirred for an additional 2 h at the same temperature. The solvent was evaporated under reduced pressure, and the residue was purified by flash chromatography as indicated in each case.

### General Procedure for the Acetylation of the Ribofuranosyl Amides

Ac_2_O (6 equiv.), pyridine (6 equiv.) and a catalytic amount of *N,N*-dimethylaminopyridine were added, at room temperature, to a solution of substrate (1 equiv.) in dry CH_2_Cl_2_ (0.1 M). The solution was stirred for 24 h and then was concentrated in vacuo. The residue was dissolved in AcOEt and washed with aqueous 5% HCl, aqueous 5% NaHCO_3_ and water. The organic layer was dried over Na_2_SO_4_ and concentrated. The crude was then purified by flash chromatography (hexane:AcOEt, 80:20 to 50:50). Purification afforded products with spectroscopic data coherent with the fully characterized compounds published by Filippov and coworkers [8].

*N^δ^-(5-tert-Butyldiphenylsilyl-α-D-ribosyl)-N^α^-benzyloxycarbonyl asparagine benzyl ester* (**12α**). The crude product was purified by flash chromatography (CHCl_3_/MeOH 95:5). yield = 60%. ^1^H-NMR (CD_3_OD, 25 °C): *δ* = 7.71–7.64, 7.42–7.24 (3 × m, 20H, Arom.), 5.73 (d, *J*_1,2_ = 4.8 Hz, 1H, H-1), 5.15, 5.07 (2 × s, 2H, CH_2_Bn, CH_2_Cbz), 4.65 (m, 1H, CH α-Asn), 4.25 (t, *J* = 5 Hz, 1H, H-3), 4.15 (t, *J* = 4.8 Hz, 1H, H-2), 3.97 (m, 1H, H-4), 3.72 (dd, *J*_4,5_ = 3.2 Hz, *J*_gem_ = 11.3 Hz, 2H, H-5 H-5’), 2.84 (m, 2H, CH_2_ β-Asn), 1.04 (s, 9H, t-Bu). ^13^C-NMR (CD_3_OD, 25 °C): 172.9, 172.4 (CO α-Asn, CO γ-Asn), 158.6 (CO Cbz), 138.2 (Cq Arom.), 137.3 (Cq Arom.), 136.9, 136.8 (Arom.), 134.6, 134.4 (Cq Arom.), 131.1, 131.0, 129.7, 129.6, 129.5, 129.5, 129.3, 129.3, 129.0, 128.8, 128.4, 128.1 (Arom.), 84.7 (C-4), 81.9 (C-1), 72.8 (C-3), 72.1 (C-2), 68.4, 67.9 (CH_2_ Bn, CH_2_ Cbz), 65.3 (C-5), 52.4 (CH, α-Asn), 38.9 (CH_2_, β-Asn), 27.4 (CH_3_, t-Bu), 20.2 (Cq, t-Bu). FT-ICR (ESI) calcd. for C_40_H_46_N_2_O_9_Si [M+Na]^+^ 749.29726; found 749.29721.

*N^δ^-(5-tert-Butyldiphenylsilyl-α-D-ribosyl)-N^α^-benzyloxycarbonyl glutamine benzyl ester* (**13α**). The crude product was purified by flash chromatography (CHCl_3_/MeOH 95:5). yield = 76%. ^1^H-NMR (CD_3_OD, 25 °C): *δ* = 7.71–7.64, 7.43–7.23 (3 x m, 20H, Arom.), 5.72 (d, *J*_1,2_ = 4.6 Hz, 1H, H-1), 5.15, 5.06 (2 × s, 2H, CH_2_Bn, CH_2_Cbz), 4.25 (m, 1H, CH α-Gln), 4.23 (t, *J* = 4.9 Hz, 1H, H-3), 4.15 (t, *J* = 4.8 Hz, 1H, H-2), 3.95 (m, 1H, H-4), 3.70 (dd, *J*_4,5_ = 3.2 Hz, *J*_gem_= 10.2 Hz, 2H, H-5 H-5’), 2.35 (m, 2H, CH_2_ γ-Asn), 2.13 (m, 1H, CH_2_ β-Asn), 1.95 (m, 1H, CH_2_ β-Asn), 1.02 (s, 9H, t-Bu). ^13^C-NMR (CD_3_OD, 25 °C): 136.7, 136.6 (Arom.), 134.6, 134.4 (Cq Arom.), 130.8, 130.8, 129.7, 129.5, 129.4, 129.1, 128.9, 128.8, 128.7 (Arom.),84.5 (C-4), 81.6 (C-1), 72.7 (C-3), 71.9 (C-2), 67.9, 67.6 (CH_2_ Bn, CH_2_ Cbz), 65.0 (C-5), 55.1 (CH α-Gln), 33.3 (CH_2_ β-Gln), 28.0 (CH_2_ γ-Gln), 27.3 (t-Bu), 22.9 (Cq t-Bu). FT-ICR (ESI) calcd. for C_41_H_48_N_2_O_9_Si [M+Na]^+^ 763.31291; found 763.31298.

## 4. Conclusions

In conclusion, we have described an improved synthesis of the building blocks **4α** and** 5α**: application of the Staudinger Traceless Ligation protocol allowed to obtain the ribosylated amino acids from **2** in good yields and with excellent selectivity for the α anomer. This represents a major advantage over pre-existing synthesis of these glycosyl aminoacids [8,16,17] and should stimulate further research in the field of ADP-ribosylation.
